# Femoral Head-Neck Translation Ratio Is a Measurement of the True Deformity of Slipped Capital Femoral Epiphysis

**DOI:** 10.7759/cureus.14133

**Published:** 2021-03-26

**Authors:** Panagiotis V Samelis, Hara Komari, Evangelos Triantafyllou, Zoi Fryda, Christos Loukas, Flourentzos Georgiou, Eleni P Sameli, Olga Savvidou, Andreas Mavrogenis, Panagiotis Koulouvaris

**Affiliations:** 1 Orthopaedics, Children’s General Hospital Panagiotis & Aglaia Kyriakou, Athens, GRC; 2 Orthopaedics, Orthopaedic Research and Education Center, Attikon University Hospital, Athens, GRC; 3 Second Orthopaedic Department, KAT Trauma Hospital of Athens, Athens, GRC; 4 Internal Medicine, Operations Center, National Public Health Organization, Athens, GRC; 5 Orthopaedics, Attikon University Hospital, Athens, GRC

**Keywords:** slipped, capital, femoral, head, epiphysis, neck, remodeling, scfe, fhntr, offset

## Abstract

A new method to quantify proximal femoral head-neck deformity in slipped capital femoral epiphysis (SCFE) is presented. In SCFE the femoral head slips posteriorly and inferiorly relative to the femoral neck. The distance of the femoral head center from the femoral neck axis (center-axis distance, CAD) represents the severity of the post-slip deformity. CAD is calculated on the anteroposterior and the frog-lateral pelvis views. It is shown that CAD is only a function of the femoral head-neck offset difference on both sides of the femoral neck. The percentage of CAD relative to the diameter of femoral neck is the femoral head-neck translation ratio (FHNTR) on the respective x-ray projection. Measurements on radiographs of 37 patients with history of unilateral SCFE were performed. The asymptomatic contralateral hips were used as controls. On the anteroposterior pelvis view, mean FHNTR was -12.2% and -4.3% for the affected and asymptomatic contralateral hips, respectively (paired t-test, p < .01), indicating inferior translation of the femoral head relative to the femoral neck. On the frog-lateral view, mean FHNTR was -21.1% and -6.5% for the affected and the contralateral hips, respectively (paired t-test, p < .01), indicating posterior translation of the femoral head relative to the femoral neck. There is a moderate inverse correlation between FHNTR on the frog-lateral pelvis view and Southwick's slip angle (Pearson correlation coefficient r = -0.679, p < .001). FHNTR on two radiological planes (anteroposterior and frog-lateral) is a simple measurement of the posteroinferior translation of the femoral head relative to the femoral neck in SCFE. It is a measurement of the true deformity of the proximal femur in SCFE. Calculation of FHNTR may be applicable to classify SCFE, to monitor femoral head-neck remodeling after slip stabilization, to describe the femoral head-neck relation in healthy individuals, and to monitor femoral head-neck changes secondary to other hip pathology, such as Perthes disease or developmental dysplasia of the hip.

## Introduction

Slipped capital femoral epiphysis (SCFE) is the most frequent non-traumatic cause of a painful limp in the adolescent. It affects predominantly the left hip of boys [1]. Half of the cases present the adiposogenital phenotype (obesity in combination with hypogonadism) indicating underlying endocrine disorder [1]. Prompt diagnosis and treatment are the only way to prevent slip exacerbation and to avoid femoroacetabular impingement (FAI) and early-onset hip osteoarthritis [1].

In SCFE, gradual external rotation and proximal migration of the femoral neck relative to the stably seated in the acetabulum capital femoral epiphysis result in slippage of the capital femoral epiphysis in two directions relative to the femoral neck: posteriorly and inferiorly [2-4]. Diagnosis is not always easy, especially in the presence of subtle symptoms or in case of lack of clinical suspicion by the examiner [5]. Missed diagnosis and delayed treatment of SCFE are reported worldwide, leading to poor outcomes of the disease [5]. Diagnosis of SCFE is based on plain radiographs of the hips.

Klein (1952) introduced the frog-lateral (FL) pelvis view for the diagnosis of SCFE. Less intersection of the capital femoral epiphysis by a line tangential to the anterior (FL pelvis view) or the superior (anteroposterior [AP] pelvis view) margin of the femoral neck (the Klein’s line), compared to the contralateral side, is diagnostic of SCFE [2]. The FL pelvis x-ray view is the examination of choice to make the diagnosis [5]. Klein used the absolute displacement of the capital femoral epiphysis on the AP and/or the FL pelvis x-ray view to decide treatment [2]. He recommended open reduction in slips greater than 1 cm and in situ pinning for less displacement [2].

Wilson (1965) classified slip severity by measuring the percentage of the displacement of the femoral head relative to the femoral neck on the AP or the lateral (not FL) pelvis view: Slips less than 1/3 of the femoral neck were deemed mild slips; slips greater than 1/3 and less than 1/2 were deemed moderate slips; and slips greater than 1/2 were deemed severe slips [4]. Wilson recommended in situ stabilization for all mild and moderate slips. The reproducibility of this classification was low, and this method was not adopted in the clinical setting [1]. Interestingly, one year before Wilson, Dunn recommended his osteotomy for slips >1/3 of the diameter of the femoral neck [6].

A few years later, Southwick (1967) described the slip angle as the difference of the head-shaft angle between the affected and the healthy hip in children and adolescents with SCFE. The head-shaft angle is measured on the FL pelvis view [3]. The Southwick classification [3], modified by Boyer, classifies slip severity into three types: <30 degrees, 30-50 degrees, >50 degrees for mild, moderate, and severe slips, respectively [7].

The scientific validity of the slip angle has been challenged [8]. In fact, the slip angle does not describe the actual pathology of the slip, because both structures (femoral head and femoral shaft), which form this angle, lie on different anatomical planes, separated by the femoral neck. These structures are projected on a two-dimensional x-ray image [9]. Nevertheless, the slip angle has been widely adopted by surgeons to classify SCFE.

However, it seems that slip classification using the slip angle is not helpful to choose between available treatment options: in situ pinning, in situ pinning with simultaneous arthroscopic osteochondroplasty, or the modified Dunn procedure. Some authors suggest that treatment should be decided after grading the slip as slight or severe by judging its radiographic appearance, without measuring at all [8]. Other authors recommend in situ stabilization for mild and moderate slips and open procedures for severe slips [10], while others support that moderate slips behave as severe slips and should receive osteotomy [11,12]. Hence, we read the monotonous repetition by almost all surgeons that in situ stabilization is the gold standard initial treatment for SCFE of any severity [1]. Furthermore, it has been supported that slip severity does not correlate with the risk of FAI after in situ stabilization [10,13]. Even mild slips are not spared from FAI and receive late hip arthroscopy [14,15]. Mamisch et al. points out that the slip angle and the head-neck offset should be assessed concomitantly to conclude the severity of post-slip FAI [11]. Thus, it seems that SCFE classifications have reached a dead end, because none is able to provide a clear recommendation for treatment different than in situ stabilization.

A new method to measure post-slip deformity is described. The distance of the femoral head center from the femoral neck axis (center-axis distance, CAD) is used to quantify the severity of the post-slip deformity. CAD is calculated on the AP and the FL pelvis view as a function of the femoral head offset difference on both sides of the femoral neck.

## Materials and methods

The records of 37 adolescents with unilateral SCFE (22 male, 15 female), 11-13-year-old, treated between 2011 and 2020, were retrospectively reviewed. According to the Loder classification, 34 slips were stable, three were unstable. According to the Southwick classification, 19 slips were mild, 13 were moderate, and five were severe slips. Twenty two slips affected the left hip. All hips underwent in situ stabilization, either with one cannulated screw (13 cases) or with two Steinman pins (24 cases). The asymptomatic contralateral hips did not undergo prophylactic fixation.

Standard AP and FL pelvis views were obtained in all patients with a stable slip. In the three patients with an unstable hip, only an AP pelvis view could be ordered on patient admission. In these patients, measurements were made on the immediate postoperative AP and FL views, which were taken after in situ fixation of the slip. Immediate postoperative x-rays were also used in patients with stable slips, when the initial FL x-ray views were deemed unsuitable for calculations, probably because of painful limitation of hip abduction.

A hypothetical model

**Figure 1:**
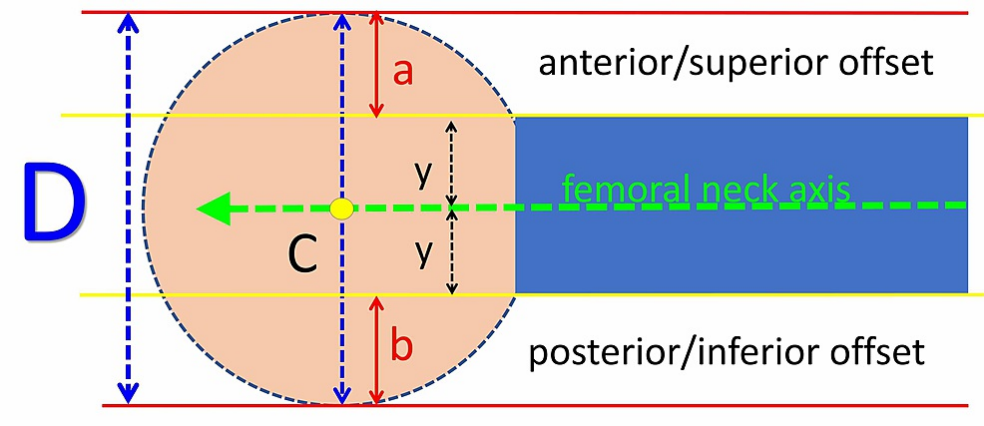
A hypothetical model of the normal femoral head-neck relation: The femoral head is considered a sphere and the femoral neck a cylinder. C: The center of the femoral head; D: the diameter of the femoral head (blue-dotted arrow); green arrow: the femoral neck axis; a: anterior/superior head-neck offset on the frog-lateral/anteroposterior pelvis x-ray, respectively; b: posterior/inferior head-neck offset on the frog-lateral/anteroposterior pelvis x-ray, respectively; y: the radius of the femoral neck.

Measurements are based on the following hypothesis: The femoral head is considered a sphere and the femoral neck a cylinder. In normal hips, it is assumed that the center of the femoral head (C) lies on the extension of the femoral neck axis. The femoral head-neck offset (a, b) on the AP and the FL pelvis views is considered equal on both sides of the femoral neck (Figure 1).

Calculation of the Femoral Head-Neck Translation Ratio

It is assumed that, secondary to hip pathology, the femoral head has slipped relatively to the femoral neck. The center of the femoral head has moved relative to the femoral neck axis from point C to point C’. The amount of slippage of the capital femoral epiphysis center relative to the femoral neck axis (the distance CC’) is the center-axis distance (CAD). CAD is calculated by solving the following equation:

$$a+y-x=x+y+b \Leftrightarrow a-x=x+b \Leftrightarrow a-b=2x \Leftrightarrow x=(a-b)/2$$

**Figure 2:**
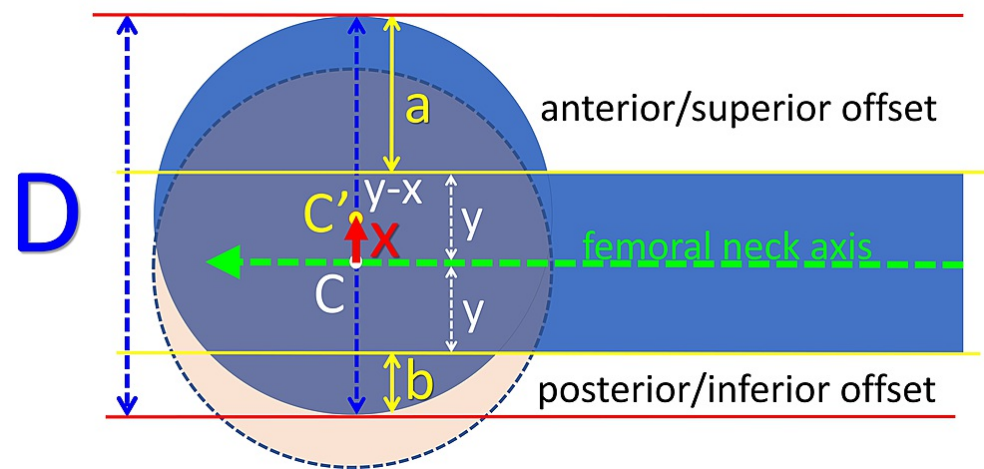
Calculation of the center-axis distance. C and C': The center of the femoral head before and after slippage, respectively; D: the diameter of the femoral head (blue-dotted arrow); green arrow: the femoral neck axis; a: anterior/superior head-neck offset on the frog-lateral/anteroposterior pelvis x-ray, respectively; b: posterior/inferior head-neck offset on the frog-lateral/anteroposterior pelvis x-ray, respectively; y: the radius of the femoral neck; x: the center-axis distance (CAD, red arrow).

Both parts of this equation represent the radius of the femoral head; y is the radius of the femoral neck, and x represents CAD (Figure 2):

Consequently,

$$CAD = (a-b)/2$$

Interestingly, CAD depends only on the difference (a - b) of the offset of the femoral head on both sides of the femoral neck and not on the radius or shape of the femoral head or neck.

To deal with different magnifications of the available x-ray projections and to avoid anthropometric variations, CAD is expressed as a percentage (%) of the diameter of the femoral head (D). This percentage is the femoral head-neck translation ratio (FHNTR):

$$FHNTR= (CAD/D)*100$$

Description of the technique in the clinical setting

**Figure 3:**
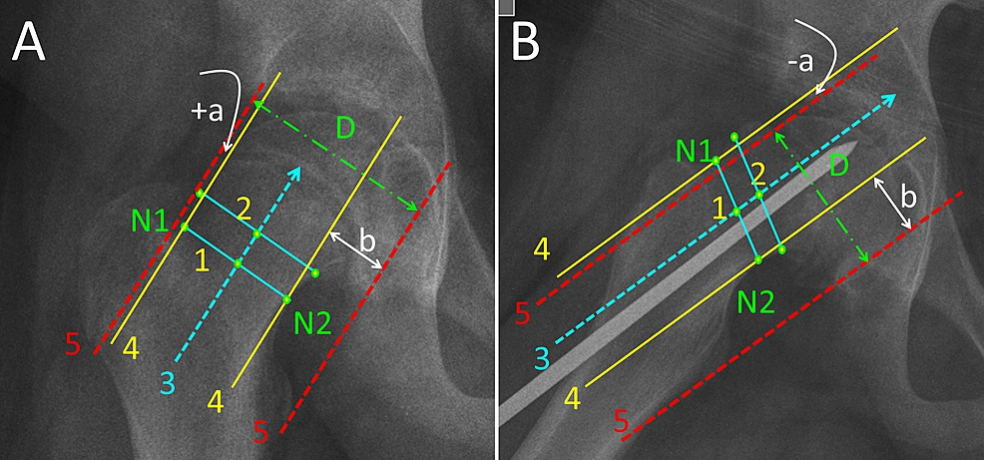
Femoral head-neck translation ratio (FHNTR) measurement on the anteroposterior (A) and the immediate postoperative frog-lateral (B) pelvis projection of a 12-year-old boy with slipped capital femoral epiphysis of the right hip, stabilized in situ with one non-threaded 6-mm stainless steel pin. N1, N2: The points defining the narrowest part of the femoral neck; line 1: connects points N1, N2; line 2: 1.5-2 cm proximal and parallel to line 1; line 3: the axis of femoral neck, through the midpoints of lines 1 and 2; line 4: parallel to line 3, tangential to points N1 and N2; Line 5: parallel to line 3, tangential to the top of the convexity on both sides of the femoral neck; D: the diameter of the femoral head (green interrupted arrow). The distance between lines 4 and 5 on either side of the femoral neck is the femoral head-neck offset: (a) is the superior/anterior head-neck offset on the anteroposterior/frog-lateral pelvis x-ray, respectively; (b) is the inferior/posterior head-neck offset on the anteroposterior/frog-lateral x-ray, respectively. When the slippage has progressed to a point, where line 5 (femoral head) is below line 4 (femoral neck isthmus), as seen in Figure 3B, the superior/anterior head-neck offset is deemed negative (-a).

FHNTR is measured on the pelvis x-ray (AP and FL) according to the following steps (Figure 3):

The first step is to define the femoral neck axis on the pelvis x-ray (AP or FL). Points N1 and N2 represent the narrowest part of the femoral neck (isthmus). A line (line 1) connecting points N1 and N2 is drawn. A second line (line 2) about 1.5 to 2 cm (through a healthy part of the femoral neck) proximal and parallel to line 1 is drawn. A line (line 3) that traverses the midpoints of lines 1 and 2 defines the femoral neck axis.

The next step is to draw two pairs of lines, parallel to the femoral neck axis (lines 4 and 5). Lines 4 are tangential to the femoral neck through points N1 and N2. Lines 5 are tangential to the femoral head contour. The distance between lines 4 and 5 on either side of the femoral neck represents the superior/anterior (a) and the inferior/posterior (b) femoral head-neck offset on the AP/FL view, respectively.

Paired samples t-test was used to compare measurements between the affected and the asymptomatic contralateral hips. Pearson’s correlation coefficient was used to assess the relation between FHNTR and the Southwick’s slip angle.

## Results

**Figure 4:**
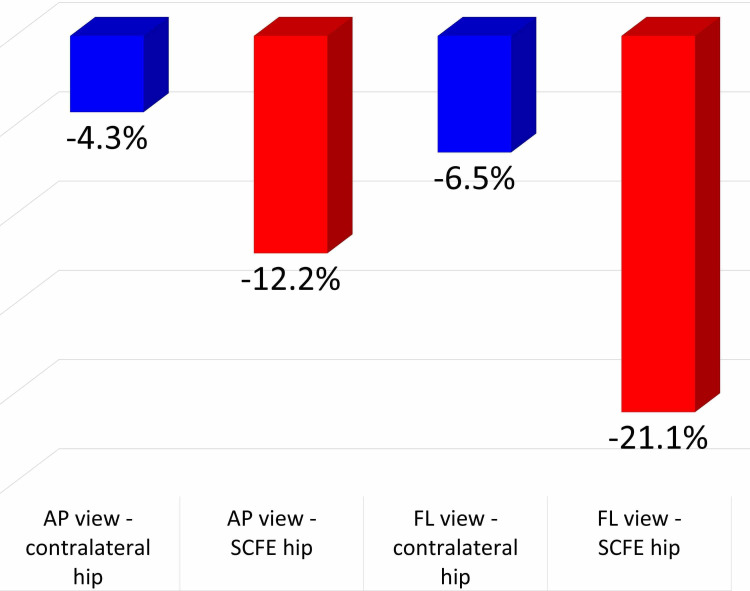
The femoral head neck translation ratio (%) on the AP and FL pelvis x-ray views at SCFE diagnosis for the asymptomatic contralateral and the SCFE hip. AP view: Anteroposterior view; FL view: frog-lateral view; SCFE, slipped capital femoral epiphysis.

On the FL view, mean FHNTR was -21.1% (SD = 10.5%) for the SCFE and -6.5% (SD = 4.1%) for the asymptomatic contralateral hips, respectively (p < 0.01). FHNTR on the FL view is negative, indicating posterior translation of the femoral head relative to the femoral neck in SCFE and asymptomatic contralateral hips. On the AP pelvis view, mean FHNTR was -12.2% (SD = 8.9%) for the SCFE and -4.3% (SD = 2.7%) for the asymptomatic contralateral hips, respectively (p < 0.01). FHNTR on the AP view is also negative, indicating inferior translation of the femoral head relative to the femoral neck in SCFE and asymptomatic contralateral hips (Figure 4).

Interestingly, even in asymptomatic (contralateral) hips, the femoral head center is displaced posteriorly/inferiorly relative to the neck axis (negative FHNTR) on the FL/AP view, respectively. In SCFE hips the femoral head center is further displaced in a posteroinferior direction compared with asymptomatic hips (p < .01).

There is a moderate negative correlation between the slip angle (FL view) and FHNTR on the FL view. Both measurements reflect posterior slippage of the capital femoral epiphysis (Pearson’s correlation coefficient, r = -0.679, p < .001) and a weak negative correlation between the slip angle and FHNTR on the AP view (inferior slippage, r = -0.495, p < .001).

## Discussion

The femoral head-neck deformity in SCFE has two aspects: the primary slip of the skeletally immature patient and the late, post-slip deformity at skeletal maturity. A late post-slip deformity leads to cam-type FAI and early hip degeneration [1]. Crucial for any treatment is the classification of the primary or the late deformity.

It is difficult to classify the true slip of SCFE on plain radiographs, because the capital femoral epiphysis slips in two directions relative to the femoral neck, posteriorly and inferiorly [4]. Several methods have been described to quantify this deformity. Additional measurements have been proposed to assess the risk of post-slip-related pathology, such as FAI. Common aim of these attempts was to set indications for the type and timing of surgical intervention.

Murray (1965) studied the pistol-grip deformity of the hip as an underlying cause of idiopathic hip osteoarthritis. He introduced the femoral head ratio (FHR). According to Murray, the femoral neck axis bisects the femoral head in two parts, the superior and the inferior. FHR is the inferior part divided by the superior part. A value greater than 1.35 indicates a pathologic pistol-grip deformity, secondary to SCFE or other unknown hip pathology [16].

Hansson et al. (1987) described a radiological method by which, as they supported, it was possible to detect a previous SCFE after physeal closure [17]. They focused on the displacement of the femoral head center relative to the femoral neck axis on the FL pelvis view. Their method to determine the femoral neck axis according to the distance from the calcar femorale is interesting, but it may be affected by anthropometrical variation or magnification issues of the x-rays. Furthermore, they define the center of the femoral head as the point, where the axis of the femoral head (the vertical bisector of the line, which spans the margins of the femoral head) transects the femoral head. However, after physeal closure or in chronic cases with a deformed femoral head, it is impossible to define the femoral head center.

Kallio et al. (1991) graded slip severity using ultrasound measurements of the head-neck junction. Translation of the capital femoral epiphysis greater than 2 mm was considered diagnostic of SCFE. Slips between 2-7 mm, 8-11 mm, and >12 mm were considered mild, moderate, and severe, respectively [18]. This method is applicable only at SCFE diagnosis (open physis) [18].

Ito et al. (2001) described in an MRI study the diminished offset of the anterolateral femoral head-neck junction as the underlying etiology of cam-type impingement that causes repetitive microtrauma to the acetabular labrum. This was frequently observed in hips with SCFE but could be detected in apparently normal hips as well [19]. Eijer et al. measured the femoral head-neck offset on the cross-table lateral view of the hip. They found that the anterior head-neck offset in normal (asymptomatic) hips was approximately 1 cm, and the anterior offset ratio (anterior offset divided by the diameter of the femoral head) was about 20% [20].

The alpha angle [21] is a very useful measurement of the femoral head-neck deformity, especially in cases of post-slip cam-type FAI. The alpha angle was originally measured on specific MRI cuts of the hip, but nowadays surgeons routinely use plain x-rays to measure it [22]. This measurement has some shortcomings. Regarding SCFE, the alpha angle has been measured in cases with established FAI, not in first diagnosed SCFE cases. Measurement is possible after the femoral head center has been identified on the x-ray or the MRI image. This is not always easy as the femoral head may be deformed, especially in late cases. Furthermore, Noetzli calculated the confidential limits of the alpha angle of patients with FAI and questioned whether an alpha angle greater than 50 degrees may be a cut-off point above which cam-type FAI occurs [21]. Given that symptomatic FAI depends on many factors and not only on femoral head-neck morphology, Noetzli’s alpha angle is useful not to make diagnosis of FAI in an asymptomatic patient (incidental finding) but to confirm cam-type FAI and assess the outcome of surgical (open or arthroscopic) treatment in patients with symptoms of FAI. Compared to FHNTR, the alpha angle is applicable only in late cases of SCFE or in other chronic hip pathology.

Gosvig et al. introduced the triangular index for the assessment of femoral head asphericity and for the prediction of the risk of FAI. The triangular index is interrelated with Noetzli’s alpha angle. This index is measured after the femoral head center has been defined on plain x-rays using the Mose’s concentrical circles, provided that the femoral head is spherical [23].

Toogood et al. (2009) first stated that although the spherical femoral head is thought to be centered on the femoral neck axis, this may not represent normal anatomy [24]. They studied femoral head translation relative to femoral neck by measuring the head-neck offset on either side of the femoral neck on the AP (superior offset [SOS] and inferior offset [IOS]) and the lateral views (anterior offset [AOS] and posterior offset [POS]) in normal cadaveric adult femora. The next step was to calculate the SOS/IOS ratio (AP view) and the AOS/POS ratio (lateral view). A ratio equal to one represents a centered femoral head on the femoral neck axis. A ratio greater than one on the AP view means that the superior offset is greater than the inferior offset, indicating superior translation of the femoral head center relative to the femoral neck axis and vice versa. A ratio greater than one on the lateral view means that the anterior head-neck offset is greater than the posterior offset, indicating anterior translation of the femoral head center relative to the femoral neck axis and vice versa. They found that in normal adults the femoral head is translated anteriorly (mean anterior/posterior offset ratio = 1.14) and inferiorly (mean superior/inferior offset ratio = 0.90) relative to the femoral neck axis [24]. On the contrary, Nemtala et al. found a mean anterior/posterior offset ratio of 0.56 ± 0.1 for a symptomatic FAI group compared with 0.9 ± 0.2 of the control group, indicating a centered position of the femoral head on the femoral neck in normal individuals and posterior translation of the femoral head on the femoral neck in FAI patients [25]. They did not measure the superior/inferior offset ratio on the AP view [25].

Wensaas et al. (2012) studied the post-slip proximal femoral head-neck deformity by measuring Murray’s FHR, Noetzli’s alpha angle in two planes (AP and FL views), and the anterior head-neck offset ratio on the FL view after in situ stabilization of SCFE. They concluded that only the alpha angle was predictive of a poor outcome on the long term [22].

Murgier et al. (2013) described the lateral view head-neck index (LVHNI). LVHNT is a measurement of the femoral head center distance from the femoral neck axis on the FL pelvis view. They aimed to assess post-slip head-neck deformity. Their method was quite complicated. They concluded that an index greater than 9% indicates post-slip deformity of the femoral head [26].

These methods have certain weaknesses. Some measure proximal femoral deformity in one plane (AP or FL plane), while others measure femoral head displacement relative to the femoral neck axis after manually defining the femoral head center on the lateral x-ray view.

Other studies used computerized tomography (CT) scans of the hip. Cohen et al. (1986) recognized the shortcomings of most radiologic classifications of SCFE due to the difficulty to obtain standardized FL views in slips of higher severity. They showed that CT measurement of the head-neck angle (HNA) is more accurate and less position-dependent than the radiologic head-shaft angle measurement (Southwick method) or the measurement of the percentage of displacement of the femoral head relative to the femoral neck (Wilson method) [8]. However, a routine CT scan implies increased radiation exposure and cannot be routinely recommended for SCFE patients.

Monazzam et al. (2013), using CT scans of the hip, described the axial oblique HNA (AOHNA). AOHNA is a measurement of the angulation of the capital femoral epiphysis relative to the femoral neck at the plane of maximum deformity. In this method, the CT plane runs parallel to the femoral neck (oblique to the axial plane of the patient) through the center of the femoral head [27]. Compared to the Southwick’s slip angle on plain x-rays, the AOHNA is more representative of the severity of the slip, and the authors suggest that AOHNA should be taken into account in selecting the type of surgery or for the diagnosis of suspected cases of SCFE [27].

Cooper et al. (2014) stated that the available classifications of SCFE underestimate the severity of the slip. They measured femoral head-neck angular deformity using a combination of plain radiography and CT [28]. The femoral head-neck deformity on the coronal plane is assessed by the HNA on the AP pelvis view. The femoral head-neck deformity on the axial plain is assessed by measuring the HNA on the axial CT. Using the Pythagorean theorem, the authors combine the HNA from the coronal (AP) and axial (CT) planes and calculate the oblique plane deformity angle (OPDA). OPDA represents, according to the authors, the true magnitude of the deformity of the femoral head-neck junction in SCFE. They recommend the modified Dunn procedure if OPDA is greater than 50 degrees regardless of slip stability [28].

Bland et al. (2019) measured 3D translational and angular deformity in SCFE using CT scans [29]. The authors presented the theta angle, which is the angle formed between the femoral neck axis and the epiphysis vector. They considered the femoral neck a perfect cylinder and the femoral head a perfect sphere. In SCFE hips, they found a (mean) posterior translation of 9.1 mm, a medial translation of 3.1 mm, and an inferior translation of 7.4 mm. The theta angle was significantly greater in SCFE hips (46.5°) compared with asymptomatic contralateral (control) hips (13.7°) and hips of individuals without hip disease (normal hips) (11.7°) hips. There was no significant difference of the theta angle between control and normal hips. The authors support that the theta angle is the true angle of deformity in SCFE hips [29].

There is no doubt that the angular (theta angle) and the translational deformity measurement by Bland et al. is probably the most precise method to describe and quantify the femoral head-neck deformity in SCFE hips. Splinting of the hip secondary to pain does not affect the CT scans. However, the authors admit that their method is complicated, and it is not expected to be adopted in clinical practice [29].

FHNTR is a different aspect of the method proposed by Toogood et al. [24]. Main advantage of FHNTR is that it is a direct measurement of femoral head center translation, whereas the method of Toogood et al. is an estimation of the direction of the translation. FHNTR is conceptually similar to LVHNI, proposed by Murgier et al. [26]. Both methods measure the distance of the femoral head center from the femoral neck axis. Main advantage of FHNTR is that it is calculated without the prerequisite to locate the femoral head center on the respective x-ray projection of the hip.

FHNTR measurement complies with the results of the study of Bland et al. [29]. Both methods describe the posteroinferior translation of the capital femoral epiphysis relative to the femoral neck, which is observed in the typical SCFE cases. However, compared to the theta angle, FHNTR does not provide information about mediolateral translation of the femoral head center. Nevertheless, FHNTR is measured using only plain radiographs (AP and FL), which are routinely taken in SCFE patients.

FHNTR is a function of the femoral head-neck offset on either side of the femoral neck and not of the radius of the femoral head or femoral neck. There is no need to define the femoral head center to calculate FHNTR. In case of a deformed femoral head, as is frequently seen in case of chronic slips, it may be difficult or impossible to define femoral head center on plain radiographs [25]. Measurement of the head-neck offset on both sides of the femoral neck is easy and reproducible after the femoral neck axis has been defined following precise steps.

A main limitation of the present study was the difficulty to obtain standard AP and FL pelvis projections on patient admission, probably due to painful splinting and subsequent diminished abduction of the hip. In case of improper initial radiographs, the first postoperative x-ray view of the pelvis was used for calculations. Such a radiograph was usually available within one-two weeks postoperatively. A second limitation is that this study is practically a retrospective radiological review with poor correlation of the measurements with the patient’s complaints. All patients were treated with in situ stabilization, irrelevant of slip severity.

FHNTR on the FL pelvis projection correlates moderately with the slip angle (a low Pearson's correlation coefficient). This is expected, because the slip angle does not correspond to the true level of the deformity, which is the femoral neck physis. The slip angle is formed between two structures, which lie on different planes (femoral head and femoral shaft). On the contrary, FHNTR is a measurement of the slippage at the true level of the deformity.

FHNTR may be useful to define normal values of the femoral head-neck junction (healthy individuals), to describe stages of severity of SCFE, and to study femoral head-neck remodeling after treatment of SCFE. FHNTR is independent from femoral head and neck radius and shape; therefore, it may be suitable for the study of other progressive hip disease, such as developmental dysplasia of the hip or Perthes disease. Long-term follow-up studies are needed to correlate FHNTR with the risk of cam-type FAI. This correlation might lead to indications for surgical treatment of the primary slip other than in situ stabilization.

## Conclusions

Many attempts have been made to describe the femoral head-neck deformity in SCFE patients. The slip angle has been widely adopted to classify SCFE; however, it does not reflect the true anatomic deformity of SCFE and does not correlate well with the risk for FAI, nor does it provide a clear recommendation for other than in situ stabilization surgical treatment. On the other hand, novel surgical techniques, such as hip arthroscopy and the modified Dunn procedure, have raised the need for clearer indications of these techniques for the primary treatment of SCFE. FHNTR is a simple method to quantify the true femoral head translation relative to the femoral neck in patients with SCFE. FHNTR is a function only of the femoral head-neck offset difference of both sides of the femoral neck. Measurements are possible even in case of a deformed-aspherical femoral head. FHNTR is a more accurate measurement of both the primary and the late deformity in SCFE hips, compared to currently available methods. Quantification of the true deformity may help to decide the type of surgery (in situ fixation, adjunct arthroscopy, femoral neck osteotomy) for the primary treatment of SCFE. Furthermore, FHNTR may be useful not only to monitor femoral neck remodeling in SCFE but also to study other pathology of the growing hip, such as Perthes disease or developmental dysplasia of the hip.
